# Optimization of tenocyte lineage-related factors from tonsil-derived mesenchymal stem cells using response surface methodology

**DOI:** 10.1186/s13018-020-01623-8

**Published:** 2020-03-17

**Authors:** Soon-Sun Kwon, Hyang Kim, Sang-Jin Shin, Seung Yeol Lee

**Affiliations:** 1 Department of Mathematics, College of Natural Sciences, Ajou University, Suwon, Gyeonggi Korea; 2 Department of Orthopaedic Surgery, Ewha Womans University Seoul Hospital, Seoul, Korea; 3 Ewha Medical Research Institute, School of Medicine, Ewha Womans University, Seoul, Korea; 4 Division of Mechanical & Biomedical Engineering, Ewha Womans University, 52, Ewhayeodae-gil, Seodaemun-gu, Seoul, 03760 Korea; 5 Department of Orthopaedic Surgery, Myongji Hospital, Hanyang University College of Medicine, Seoul, Korea

**Keywords:** Tenocyte, Tonsil-derived mesenchymal stem cells, Bone marrow-derived mesenchymal stem cells, Design of experiments, Response surface methodology

## Abstract

**Background:**

In order to optimize the tenogenic differentiation of mesenchymal stem cells (MSCs), researchers should consider various factors. However, this requires testing numerous experimental settings, which is costly and time-consuming. We aimed to assess the differential effects of transforming growth factor beta-3 (TGF-β3) on the tenogenesis of tonsil-derived MSCs (T-MSCs) and bone marrow-derived MSCs (BM-MSCs) using response surface methodology (RSM).

**Methods:**

Bone marrow and tonsillar tissue were collected from four patients; mononuclear cells were separated and treated with 5 or 10 ng/mL of TGF-β3. A full factorial experimental design with a categorical factor of 0 was employed to study the effect of tension based on T-MSCs. Eighty-four trials were fitted with RSM and then used to obtain mathematical prediction models.

**Results:**

Exposure of T-MSCs and BM-MSCs to TGF-β3 increased the expression of scleraxis (SCX), tenomodulin (TNMD), decorin, collagen I, and tenascin C. Expression of most of these factors reached a maximum after 2–3 days of treatment. The model predicted that the values of the tenocyte lineage-related factors assessed would be significantly increased at 2.5 days of culture with 2.7 ng/mL of TGF-β3 for T-MSCs and at 2.3 days of culture regardless of TGF-β3 concentration for BM-MSCs.

**Conclusions:**

This study demonstrated that the RSM prediction of the culture time necessary for the tenogenic differentiation of T-MSCs and BM-MSCs under TGF-β3 stimulation was similar to the experimentally determined time of peak expression of tenocyte-related mRNAs, suggesting the potential of using the RSM approach for optimization of the culture protocol for tenogenesis of MSCs.

## Background

Tissue healing after tendon repair surgery is challenging [1] because tendons have limited self-healing potential given the scar tissue on the repaired tendon [2, 3]. Even after long periods, the structure and strength of the repaired tissue do not show full recovery, and the tissue does not return to the pre-injury state [4]. For these reasons, there have been various studies done on tendon repair using tissue engineering with mesenchymal stem cells (MSCs) [5–9].

Although bone marrow-derived, adipose tissue-derived, synovial membrane-derived, and human embryonic MSCs have all been used to differentiate into tenocytes [6, 9, 10], these stem cells are obtained through invasive procedures. Thus, there is usually a lack of an adequate number of MSCs for clinical use [11]. Tonsil-derived MSCs (T-MSCs) obtained from waste tissue after tonsillectomy represent a new source of progenitor cells [12, 13], and several studies have focused on T-MSCs as cellular therapeutic agents for various diseases [12–19]. A previous study reported that T-MSCs have a good potential of being suitable for clinical banking of stem cells [12]. This means that it might be possible to conduct tonsil tissue banking (after tonsillectomy) for autogenic MSCs grafts to be used in case of future injury or disease. In the United States, 68.7% of the patients who underwent tonsillectomy were children less than 15 years old [20]. The prevalence of tendinopathies such as rotator cuff tears increased with age [21]. Because the donor age may affect the differentiation potential of MSCs [22, 23], T-MSCs harvested when a patient is young could have an advantage in terms of tenogenic differentiation potential compared with BM-MSCs harvested at the time of treatment. If T-MSCs show non-inferior tenogenic differentiation potential compared with other cell sources, physicians can consider tonsils as a stem cell source for cellular therapeutic agents. However, protocols for tenogenic differentiation of T-MSCs have not been established, and there is a lack of studies comparing the tenogenic differentiation potential of T-MSCs with MSCs from other cell sources.

Researchers should consider various potential factors affecting tenogenic differentiation of MSCs, such as culture time and the dose of the stimulator; however, this requires numerous experimental settings whose optimization is associated with high cost and time. Response surface methodology (RSM), which is used as a part of the design of experiments (DoE), is gaining recognition as a powerful approach for optimizing conditions to produce industrially important products such as chemicals and enzymes. It allows for multiple input factors to be manipulated, determining their effect on the desired output. In the last few years, RSM has been applied to optimize and evaluate the interactive effects of independent factors in numerous chemical and biochemical processes [24]. Recently, DoE has been used to investigate the differentiation of MSCs [25]. The main advantages of this methodology are that it (1) avoids experimental bias and (2) reduces the number of experiments, leading to a rational understanding of what could be the most favorable factor combination [24, 26]. By manipulating multiple inputs at the same time, DoE can identify important interactions that may be missed when experimenting with one factor at a time.

In this study, we aimed to optimize the culture conditions for the tenogenesis of T-MSCs and bone marrow-derived MSCs (BM-MSCs) using TGF-β3, testing different concentrations and culture times.

## Materials and methods

This experimental study was approved by the institutional review board of our institution. Informed consent was obtained from all the patients or patients’ legal guardians.

### Isolation and cultivation of human MSCs

Bone marrow was collected from four patients (mean age 79.0 ± 2.2 years) and mononuclear cells were separated using the Ficoll-Paque Premium (GE Healthcare, Chicago, IL, USA) gradient method. The isolated cells were seeded at a density of 1 × 10^5^ cells/cm^2^ in a growth medium consisting of low-glucose Dulbecco’s Modified Eagle’s Medium (DMEM-LG; Hyclone, South Logan, UT, USA), 10% fetal bovine serum (FBS; Corning, Corning, NY, USA), 100 U/mL penicillin, and 100 μg/mL streptomycin. The tonsillar tissues were collected from four patients (mean age 7.6 ± 0.6 years) and minced with surgical scissors, followed by enzymatic digestion using 210 U/mL collagenase type I (Sigma, St. Louis, MO, USA) and 90 KU/mL DNase (Sigma, St. Louis, MO, USA) in DMEM-LG for 30 min at 37 °C. After filtration through a 100-μm cell strainer (BD Bioscience, Franklin Lakes, NJ, USA), the cells were washed twice with Dulbecco’s phosphate-buffered saline (D-PBS; Chembio, Seoul, Korea). Mononuclear cells were separated using the Ficoll-Paque gradient method [13] and seeded at a density of 1 × 10^4^ cells/cm^2^ in growth medium. MSCs were incubated in a 5% CO_2_ incubator with humidified air at 37 °C, and the medium was replaced every other day. After reaching 80% confluency, the cells were split at a ratio of 1:3 for BM-MSCs and 1:4 for T-MSCs. MSCs were used between passages two and four for further experiments. Cell surface antigen phenotyping was performed on BM-MSCs and T-MSCs at the second passage to characterize the immunophenotype of MSCs using BD Stemflow™ hMSC Analysis kit (BD Bioscience, Franklin Lakes, NJ, USA). The analysis kit contained pre-conjugated and pre-titrated cocktails of the International Society for Cellular Therapy-defined positive expression markers (CD105-PerCP-Cy™5.5/CD73-APC/CD90-FITC) and negative expression markers (CD45/CD34/CD11b/CD19/HLA-DR-PE). Mouse isotype antibodies served as control. Labeled cells (1 × 10^6^) were analyzed using a BD FACS LSR II SORP system (Becton Dickinson).

### Tenogenic differentiation of BM-MSCs and T-MSCs

BM-MSCs and T-MSCs were seeded at a density of 1 × 10^4^ cells/cm^2^ into 24-well plates in growth medium. After 18 h, the growth medium was removed and replaced with tenogenic differentiation media, which consisted of DMEM-LG, 10% FBS, and 50 μg/mL L-ascorbate-2-phosphate with 5 or 10 ng/mL TGF-β3 (Sigma, St. Louis, MO, USA). MSC growth medium was added to the control group. The medium was replaced three times a week.

### Isolation of RNA and quantitative real-time PCR

Total RNA was isolated daily for up to 7 days using the total RNA mini kit supplemented with DNase I (NucleoGen Biotechnology). First-strand cDNA was synthesized using SuperScript III first-strand cDNA synthesis kit (Invitrogen), and quantitative real-time PCR was performed with SensiFAST SYBR Hi-ROX kit (Bioline) using the QuantStudio 3 real-time PCR system (Applied Biosystem, Life Technologies). The relative expression level of each gene was normalized to that of 18S rRNA and calculated using the 2^−ΔΔCt^ method. The data are presented as fold changes relative to controls. The following genes were analyzed: scleraxis (SCX), tenomodulin (TNMD), decorin, collagen I/III, and tenascin C. These genes have a crucial role in the tenogenesis of MSCs [27–29]. The primers used in this study are shown in Table 1.

**Table 1:** Primers used in this study*

	Gene	Forward Primer (5'->3')	Reverse Primer (5'->3')
Adipogenic	PPARγ	GAGGCAGCAGAGGTTAACAGA	CACCGAGGCGTAAAGTACCA
	LPL	CCGCCGACCAAAGAAGAGAT	TAGCCACGGACTCTGCTACT
	FABP4	TGGGCCAGGAATTTGACGAA	CACATGTACCAGGACACCCC
Osteogenic	RUNX2	CCTACCTGAGCCAGATGACG	ATGCTGGGTGGCCTGAAAT
	ALP	GAATCTTCCCCAAGGGCCAA	CAGAATGTTCCACGGAGGCT
	BGLAP	TCCTTTGGGGTTTGGCCTAC	CCAGCCTCCAGCACTGTTTA
Chondrogenic	SOX9	AGGAAGTCGGTGAAGAACGG	AAGTCGATAGGGGGCTGTCT
	COL2	GCTCCTGCCGTTTCGCTG	ATTATACCTCTGCCCATCCTGC
	ACAN	CTTCCGCTGGTCAGATGGAC	CGTTTGTAGGTGGTGGCTGT
Tenogenic	SCX	ACAGATCTGCACCTTCTGCC	GCCACCTCCTAACTGCGAAT
	TNMD	TCCCTCAGGCTCTGGTATGA	AGGACTGAGAGACCACTGCT
	DCN	TGCCAAAGGATCTTCCCCCT	AGGTGTAAATGCTCCAGGACT
	COL1A1	AGTGGTTTGGATGGTGCCAA	GCACCATCATTTCCACGAGC
	COL3A1	TGGAGGATGGTTGCACGAAA	ACAGCCTTGCGTGTTCGATA
	TNC	ATGGGCAGACGCACCATTAG	TGTGCATCGACCTTCACAAGA
Internal control	18S rRNA	GTAACCCGTTGAACCCCATT	CCATCCAATCGGTAGTAGCG

*NCBI/ Primer-BLAST: Finding primers specific to your PCR template (using Primer3 and BLAST). Available from: https://www.ncbi.nlm.nih.gov/tools/primer-blast/ (Assessed on 15 December 2017)

### Experimental design

A full factorial experimental design with a categorical factor of 0 was employed to study the effect of tension in T-MSCs. The design comprised of three levels coded as −1, 0, and +1. In total, 18 runs were performed in duplicate to optimize the level of the chosen variables, such as the number of days in culture and amount of TGF-β3 added. For the purpose of statistical computation, two independent variables denoted as *x*_1_ and *x*_2_ were selected. The levels used in the computation were determined from preliminary experiments and are presented in Table 2.

**Table 2:** ANOVA for response surface model

MSCs	Source	Sum of squares	Mean square	*F* value	*p* value
T-MSCs	Days	17.57867	5.859557	9.98	< 0.001
	Concentration of TGF-β3	88.30324	29.43411	50.11	< 0.001
BM-MSCs	Days	37.2556	12.4185	3.68	0.013
	Concentration of TGF-β3	71.4878	23.8293	7.07	< 0.001

*MSCs* mesenchymal stem cells, *T-MSCs* tonsil-derived mesenchymal stem cells, *BM-MSCs* bone marrow-derived mesenchymal stem cells

The results were analyzed via analysis of variance (ANOVA). Multi-level factorial designs were used to estimate the response, calculated according to the following second-degree polynomial equation (1):

1 $$ Y={\beta}_o+{\sum}_{i=1}^2{\beta}_i{X}_i+{\sum}_{i=1}^2{\beta}_{ii}{X}_i^2+{\sum}_{i\ne j}{\beta}_{ij}{X}_i{X}_j $$

where *Y* is the estimated response; *β*_*o*_, *β*_*i*_, *β*_*ii*_,and *β*_*ij*_ are the regression coefficients for intercept, linearity, square, and interaction, respectively; and *X*_*i*_ and *X*_*j*_(i, j = 1, 2, i ≠ j) are the different interaction coefficients between the predicted response and independent variables in the coded values according to Table 2.

In this study, RSM combined with a full factorial design was used to investigate MSCs in tissues. By using a multi-level two-factorial design and a full range of RSM, the following parameters were optimized: SCX, TNMD, decorin, collagen I/III ratio, and tenascin C. A second-order polynomial regression model was used to generate three-dimensional response surfaces of MSCs. The regression model would provide a good explanation of the relationship between the independent variables and responses [30].

### Statistical analysis

The statistical significance among different concentrations and time points was analyzed by ANOVA, followed by Tukey’s multiple comparison test using SAS version 9.4.2 (SAS Institute, Cary, NC). All statistics were two-tailed, and *p* < 0.05 was considered significant.

## Results

### Immunophenotypic characterization of BM-MSCs and T-MSCs

Immunophenotypic surface marker analysis of BM-MSCs and T-MSCS showed the typical expression profile of human MSCs. Both MSC populations expressed CD73, CD90, and CD105, whereas they lacked expression of CD11b, CD19, CD34, CD45, and HLA-DR (Table 3). The T-MSCs used in this study showed stemness, meaning that they are at least able to differentiate into adipocytes, osteoblasts, and chondroblasts [31].

**Table 3:** Immunophenotypic markers on the cell surface of T-MSCs and T-MSCs

		BM-MSCs	T-MSCs
Positive	CD73	99.9 ± 0.05	99.9 ± 0.03
	CD90	99.6 ± 0.00	97.7 ± 1.57
	CD105	99.8 ± 0.10	99.9 ± 0.05
Negative	CD11b	1.2 ± 1.00	0.23 ± 0.03
	CD19		
	CD34		
	CD45		
	HLA-DR		

*T-MSCs* tonsil-derived mesenchymal stem cells, *BM-MSCs* bone marrow-derived mesenchymal stem cells

### Expression of tenogenic genes in MSCs under TGF-β3 stimulation

**Fig. 1:**
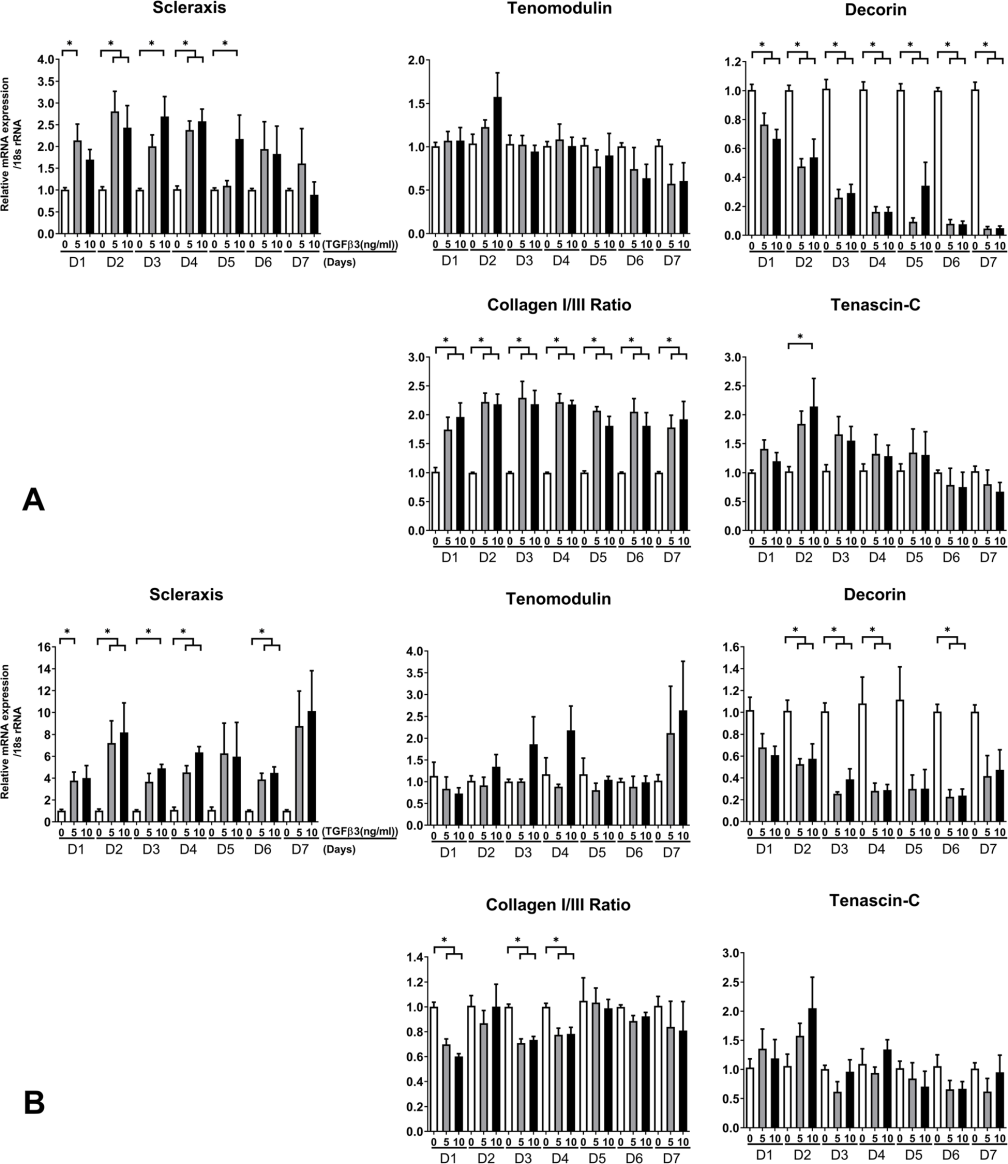
Tenogenic mRNA expression of T-MBCs (**a**) and BM-MSCs (**b**) exposed to TGF-β3. The peak expression of each mRNA differed slightly according to the culture time and TGF-β3 concentration. The collagen I to III ratio of T-MSCs increased regardless of TGF-β3 concentration, whereas the ratio in BM-MSCs decreased. (*p < 0.05, analyzed by one-way analysis of variance, followed by Tukey’s multiple comparison test)

Exposure of T-MSCs and BM-MSCs to TGF-β3 resulted in increased expression of SCX, TNMD, and tenascin C, as well as increased collagen I/III ratio. The expression of decorin was lower in both T-MSCs and BM-MSCs than in untreated MSCs. The peak expression of each mRNA varied slightly according to the culture time and the concentration of TGF-β3 (Fig. 1).

### Optimization of tenocyte lineage-related factors from T-MSCs

**Fig. 2:**
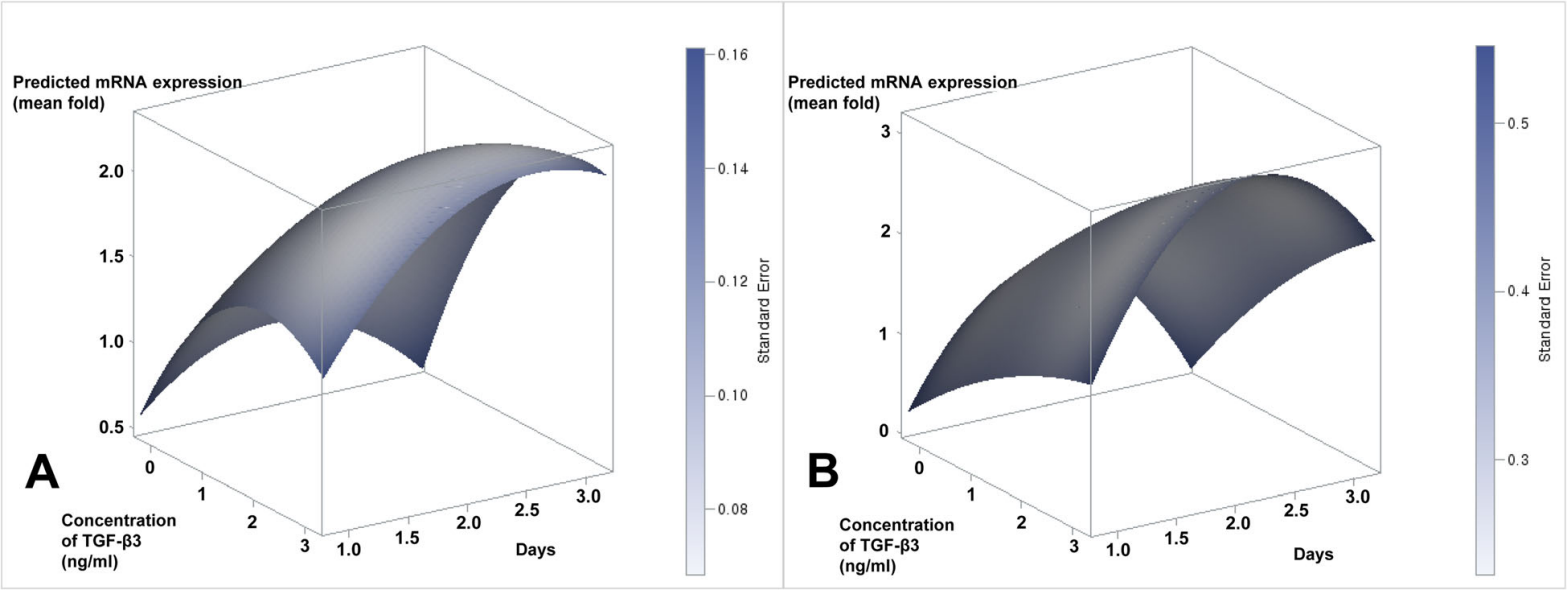
Response surface for all tenocyte lineage-related mRNAs from T-MSCs (**a**) and BM-MSCs (**b**)

The DoE used in this study allowed for the optimization of tenocyte lineage-related factors from T-MSCs and BM-MSCs under different TGF-β3 concentrations and culture times. From the DoE approach, the predicted value of SCX from T-MSCs was significantly increased at 8.4 ng/mL TGF-β3 (*p* = 0.014) and 2.3 days (55.2 h) of culture (*p* = 0.040); the expression of collagen I showed the maximum increase at 8.1 ng/mL TGF-β3 (*p* < 0.001) at 2.7 days (64.8 h) of culture (*p* = 0.036); TNMD peaked at 2.5 days (60 h) of culture (*p* = 0.011), regardless of TGF-β3 concentration; and TGF-β3 concentration affected the peak expression of decorin (*p* < 0.001) and the ratio of collagen I to III (*p* < 0.001) regardless of culture time (Table 4). For all the tenocyte lineage-related mRNAs that were assessed, the predicted value of the factors was significantly increased at 2.7 ng/mL TGF-β3 (*p* < 0.001) at 2.5 days of culture (*p* = 0.001) (Fig. 2a).

**Table 4:** Optimization of each tenocyte lineage-related factor from T-MSCs and BM-MSCs using response surface methodology

	Estimated value from T-MSCs				Estimated value from BM-MSCs			
	Time (day)	*p* value	Concentration	*p* value	Time (day)	*p* value	Concentration	*p* value
Scleraxis	2.3	0.040	8.4	0.014	2.1	0.014	3.6	0.133
Tenomodulin	2.5	0.011	31.2	0.528	2.9	0.872	0.6	0.034
Decorin	4.8	0.891	1.1	< 0.001	0.6	0.599	2.0	0.004
Collagen I/III	2.8	0.376	7.8	< 0.001	2.1	0.009	2.3	0.018
Tenacin C	2.2	0.038	7.6	0.075	1.9	0.019	0.2	0.805

*TGF-β3* transforming growth factor-beta 3, *T-MSCs* tonsil-derived mesenchymal stem cells, *BM-MSCs* bone marrow-derived mesenchymal stem cells

### Optimization of tenocyte lineage-related factors from BM-MSCs

From the DoE approach, the predicted maximum ratio of collagen I to III in BM-MSCs was significantly increased at 2.3 ng/mL TGF-β3 (*p* = 0.018) and 2.1 days of culture (*p* = 0.009); the maximum expression of SCX, collagen I, and tenascin C was affected by culture time; the expression of TNMD and decorin peaked at 0.6 ng/mL (*p* = 0.036) and 2.0 ng/mL (*p* = 0.004) of TGF-β3, respectively, regardless of culture time for the predicted peak expression of TNMD (*p* = 0.872) and decorin (*p* = 0.599) (Table 4). For all the tenocyte lineage-related mRNAs that were assessed, the predicted value of the factors was significantly increased at 2.3 days of culture (*p* = 0.004) regardless of TGF-β3 concentration (Fig. 2b).

A full factorial design is a powerful tool for understanding complex processes of tenocyte lineage-related factors in multifactor systems because it includes all possible factor combinations for each of the factors. RSM is an empirical statistical technique employed for multiple regression analysis that uses quantitative data obtained from design experiments to solve multivariate equations simultaneously.

The quadratic equation for predicting the optimum point was obtained according to the data and input variables, and then the empirical relationship between the response and independent variables in the coded units was presented based on the experimental results as follows:

$$ Y=0.06+1.14{X}_1+0.623{X}_2-0.288{X}_1^2-0.163{X}_2^2+0.105{X}_1{X}_2 $$

where *Y* is the T-MSCs, and *X*_1_ *and X*_2_ are the time in days and the concentration of TGF-β3, respectively (Table 5). The results of the ANOVA for the quadratic equation are tabulated in Table 5. The ANOVA shows whether the equation and actual relationship between response and significant variables represented by the equation are accurate. The significance of the coefficient term is determined by the values of *F* and *p*, and larger values of *F* and smaller values of *p* represent more significant terms.

**Table 5:** ANOVA for response surface quadratic model

MSCs	Source	Estimate	Standard error	*F* value	*p* value
T-MSCs	Intercept	0.064	0.337	0.04	0.849
	*X*_1_	1.143	0.352	10.50	0.0013
	*X*_2_	0.623	0.151	16.97	<.0001
	${X}_1^2$	−0.288	0.058	11.29	0.0009
	${X}_2^2$	−0.163	0.044	13.91	0.0002
	*X*_1_*X*_2_	0.105	0.039	7.08	0.0083
BM-MSCs	Intercept	−1.874	1.142	2.69	0.1029
	*X*_1_	3.534	1.195	8.76	0.0035
	*X*_2_	0.442	0.513	0.74	0.3897
	${X}_1^2$	−0.895	0.290	9.48	0.0024
	${X}_2^2$	−0.064	0.148	0.18	0.6671
	*X*_1_*X*_2_	0.117	0.134	0.76	0.3855

*MSCs* mesenchymal stem cells, *T-MSCs* tonsil-derived mesenchymal stem cells, *BM-MSCs* bone marrow-derived mesenchymal stem cells, *X*_1_, day; *X*_2_, concentration of TGF-β3

In the results, ${X}_1,{X}_2,{X}_1^2,{X}_2^2, and\ {X}_1{X}_2$were highly significant factors. The analysis of equation (1) depicted that the variables, i.e., time in days and concentration of TGF-β3, have positive effects on T-MSCs. Synergistic interactions between time and concentration were highly significant (*p* < 0.05). The results also indicated that the selected quadratic model was adequate in assuming the response variables for the experimental data.

### Three-dimensional response surface plot

Figure 2 depicts the three-dimensional response surface relationship between culture time and TGF-β3 concentration for T-MSCs and BM-MSCs. MSCs were sensitive to culture time and TGF-β3 concentration, which was consistent with the results presented in Table 5. These data indicate that the appropriate conditions would result in the highest mRNA concentration. Response surface plots as a function of two factors at a time, with the maintenance of all other factors at fixed levels, clarify both the main and interaction effects of the two factors. In the present study, the interaction effects of the variables and optimal levels of each variable were determined by the response surface graphs. The optimum values drawn from these figures were in close agreement with those obtained by optimizing the regression model equation (1).

## Discussion

In this study, we aimed to assess the effects of TGF-β3 on the tenogenesis of T-MSCs and BM-MSCs using RSM. We found that tenocyte-like cells could be successfully differentiated from T-MSCs and BM-MSCs under TGF-β3 stimulation.

Traditionally, protocol optimization for the differentiation of T-MSCs would require the consideration of various factors that influence tenogenesis, making it a resource-intensive process. We conducted this study to optimize the culture conditions for tenogenesis of T-MSCs and BM-MSCs under TGF-β3 stimulation at different concentrations and culture times using DoE. This statistical method predicted that the expression of differentiation markers would be significantly induced after 2.5 and 2.3 days in culture for T-MSCs and BM-MSCs, respectively. We validated this prediction experimentally by exposing T-MSCs and BM-MSCs to various concentrations of TGF-β3, which resulted in an increase in the expression of the differentiation markers SCX, TNMD, decorin, collagen I, and tenascin C after 2–3 days of culture.

The statistical methods used in this study, RSM and DoE, might be unfamiliar to most physicians. Here is an example of their work: while doing the interior design of a new house, the final effect will depend on various factors such as color of the walls, lights, placement of various objects, and others. The final outcome can be impacted by variation in each factor alone, or a variation in a combination of these factors at the same time. Hence, in order to do an effective job, it is important to know how each of these factors impact the final outcome, which critical factors have the most impact, and which combinations have the most significant impact. To solve this problem, the interior designer can plan and conduct some experiments. DoE is a statistical method that helps solve complicated problems and save time and cost by reducing the number of required experiments. That is, the DoE is an efficient method for planning experiments that can be analyzed to yield valid and objective conclusions with less experimental effort. The choice of DoE depends on the objectives of the experiment and the number of factors to be investigated. RSM is used as a DoE tool, used to fit models and analyze problems in which several independent variables influence a dependent variable or response. RSM has been applied for developing mathematical models in the form of multiple regression equations [32]. Recently, DoE has been used in molecular biology and tissue engineering [25]. The present study represents the first stages of research regarding tenogenesis of MSCs, and we believe that the methodology used here can contribute to future studies.

We found that the expression of all examined molecules except for decorin tended to increase after exposure of T-MSCs and BM-MSCs to TGF-β3. Our results were similar to those of a previous study [29]. Although the peak expression of each gene for T-MSCs and BM-MSCs was analyzed using DoE, the optimal culture time and concentration of TGF-β3 varied for each gene. For instance, the expression of decorin decreased as the culture progressed, whereas the expression of TNMD peaked during the 7-day culture in BM-MSCs after TGF-β3 treatment. Since the expression levels of all genes that were analyzed in this study should increase for tenogenesis of MSCs, we could not determine the optimal culture time. Although the expression of TNMD did not change significantly (Fig. 1), we could not determine the expression of TNMD after exposure of MSCs to TGF-β3 owing to the small sample size and experimental settings.

There are certain limitations to this study. First, MSCs were stimulated only by a single chemical. Several other proteins can be stimulation candidates for MSCs, although we only referred to previous studies that used TGF-β for tenogenic differentiation of MSCs [8, 33]. Therefore, different approaches would be required for different chemical stimulators. Second, we did not consider the effect of mechanical stimulation on the tenogenesis of MSCs. Several studies showed that mechanical stretching stimulates MSCs to proliferate and differentiate into tenocytes [34, 35]. Further research focusing on mechanical stimulation of T-MSCs and BM-MSCs is needed.

In summary, we optimized a protocol for the tenogenic differentiation of T-MSCs and BM-MSCs using the DoE approach. This approach was less expensive than the standard experimentation-based optimization approach. In addition, our protocol was validated experimentally. This study suggests the potential of using the DoE approach for optimization of the culture protocol for tenogenesis of MSCs.

## Data Availability

The datasets used and/or analyzed during the current study available from the corresponding author on reasonable request.

## Abbreviations

ANOVA: Analysis of variance
BM-MSCs: Bone marrow-derived mesenchymal stem cells
DMEM-LG: Low-glucose Dulbecco’s Modified Eagle’s Medium
DoE: Design of experiments
D-PBS: Dulbecco’s phosphate-buffered saline
FBS: Fetal bovine serum
MSCs: Mesenchymal stem cells
RSM: Response surface methodology
SCX: Scleraxis
T-MSCs: Tonsil-derived mesenchymal stem cells
TNMD: Tenomodulin
